# Equine dietary supplements: an insight into their use and perceptions in the Irish equine industry

**DOI:** 10.1186/s13620-018-0115-3

**Published:** 2018-01-30

**Authors:** J. M. D. Murray, E. Hanna, P. Hastie

**Affiliations:** 1 School of Veterinary Medicine, University of Glasgow Veterinary School, Bearsden Road, Glasgow, G61 1QH UK; 2 Royal (Dick) School of Veterinary Studies, University of Edinburgh, Easter Bush, Roslin, Midlothian, EH25 9RG UK

**Keywords:** Equine nutrition, Horse owners, Supplements, Survey

## Abstract

**Background:**

Nutritional supplements are frequently used by horse owners/caregivers to supplement their horse(s) diets. Some work has been done to identify the types of supplements fed and the reasons for doing so; however, this has been predominantly disciple-specific and with little focus on participants’ perceptions of supplement testing and regulation. The aim of this study was to gain an insight into the use and perceptions of equine dietary supplements in the Irish equestrian industry.

**Methods:**

An online survey was designed to ascertain the following information: demographics, types of supplements fed and reasons for use, factors that influenced respondents’ choice of supplement, where advice was sought and perceptions of testing and regulation of equine supplements

**Results:**

The survey yielded 134 responses, 70% non-professionals and 30% professionals. A greater percentage of professionals included supplements in their horse(s) diets (98%) compared to non-professionals (86%). Almost 70% of professionals fed more than two supplements, whereas 80% of non-professionals reported to feed only one supplement. Joint supplements were most commonly fed by all respondents (22%) followed by calming supplements (13%). The enhancement of performance (35%) and prevention of joint disorders (34%) were the most common reasons reported by respondents for using a supplement. Over 53% of respondents sought advice on choosing a supplement from their feed merchant, followed by their veterinarian (46%). Veterinary recommendation was given as the most influential factor when choosing a supplement by 90% of respondents, followed by cost (69%). Most (93%) respondents thought that feed supplements had to meet legal standards, with each batch analysed for quality (72%) and the supplement tested on horses before being launched on to the market (92%).

**Conclusion:**

This study has identified the main types of supplements used in the Irish equestrian industry along with the reasons for their use. However, it has also highlighted major misperceptions in how supplements are tested before being launched for sale and further work on this aspect of the findings would be beneficial.

## Background

Nutritional and health supplements are frequently used to aid in the prevention of deficiencies of vitamins, mineral and electrolytes, and/or improve health or performance. The human health market is saturated with supplements claiming to do everything from balancing the diet, improving athletic performance to mitigating health issues, and indeed the equine supplement market has followed suit with significant growth over the last 20 years. This has resulted in an overwhelming array of supplements available to horse owners/caregivers.

There has been some evaluation of the use of supplements in the USA and UK that indicate widespread use of supplements [1, 2] with common reasons for giving a supplement including to alleviate a health issue, prevent health issues occurring, modify behaviour, provide additional nutrients for performance and to balance the diet [1, 3]. Although many claim to enhance performance or alleviate health issues, there is still a paucity of evidence based studies to support these claims. Consequently, horse owners/caregivers have access to a wide range of supplements with a variety of purported benefits with little or no scientific evidence to support their effectiveness [4].

Studies have reported joint supplements and electrolytes as being most widely used in the UK and USA [1, 5, 6]. The feeding of multiple supplements has also been reported, with some horses receiving up to four different types of supplements [1]. Moreover, the selection of supplements has received some attention in recent years, with some studies investigating sources of information used by horse owners/caregivers when choosing supplements [3, 7]. Indeed, Hoffman [3] reports that horse owners often do not have sufficient knowledge to make informed decisions when selecting a supplement for their horse(s). While efforts have been made to better understand the use and perception of equine supplements, these have been limited and predominantly discipline-specific [1, 3, 6, 7]. While the foregoing studies have yielded valuable information, further information on the use and perceptions of equine supplements by a widespread population of horse owners/caregivers would be extremely useful. In terms of safety and efficacy of human supplements a high proportion of adults surveyed reported to feel confident that the supplements they use are effective and safe [8]; however, few regulations exist for human or animal nutritional supplements and in fact the majority of supplements are not tested for safety of efficacy. Despite this, there has been little work to investigate horse owners/caregivers perceptions of supplement testing and regulation. Moreover, no studies have been undertaken to assess the use and perceptions of equine supplements within the Irish equine industry, despite Ireland being one of the most densely horse populated counties in the European Union [9]. Consequently, the aim of this study was to investigate the use and perceptions of equine supplements by Irish horse owners/caregivers.

## Methods

### Participants

This study involved a survey designed to ascertain participants’ use and perceptions of equine supplements. The target population were horse owners and trainers, professional and non-professional, throughout Ireland (Republic of Ireland and Northern Ireland).

### Survey design

An 11-question online survey (Bristol Online Surveys, 2011) was designed to evaluate participants’ use and perceptions of equine supplements. The survey was promoted at many equestrian events across Northern Ireland and the Republic of Ireland, in tack shops and feed merchants and veterinary clinics. The survey mainly consisted of Likert scale questions, where there was a choice of a number of fixed alternatives followed by a small number of open questions. The survey consisted of three sections: the first section collected demographic information on respondents, including whether they deemed themselves professional or non-professional and the number of horses in their care. The second section gathered details of the types of supplements fed and reasons for feeding them. The final section gathered details on the factors influencing supplement choice and where respondents sought advice on the use of supplements and their satisfaction with that advice, along with respondents perceptions of supplement testing and regulation. Prior to completing the survey participants received information on the aims of the research and the value of their contribution.

Pre testing via a pilot survey was carried out as recommended by Robson [10]. Pilot study feedback inferred only minor modifications to question phrasing, aimed at making sure questions were interpreted correctly. Participants were informed of how the data would be stored and used as part of the introduction to the survey. Participants informed that completion of the survey indicated their consent for their responses to be used in the research. All data was provided anonymously and kept strictly confidential in accordance with the UK data protection regulations. A copy of the survey can be obtained from the corresponding author.

### Data analyses

Data were gathered in the Bristol Online Survey tool and were downloaded into an Excel spreadsheet in a coded form with a key. Quantitative data were analysed for descriptive statistics and non-parametric statistical tests using SPSS statistical software.

## Results

### Demographics

The survey yielded 134 responses. More responses were received from non-professionals (94, 70%) than professionals (40, 30%). The number of horses owned or cared for differed (*P* < 0.001) between professionals and non-professionals, with 73% of professionals caring for 5 or more horses and 82% of non-professionals caring for 1 or 2 horses.

### Supplements used

A large proportion (44%) of respondents reported to provide free access to salt, with more (*P* < 0.005) non-professionals providing free access to salt than professionals (52 versus 25%, respectively). In contrast to providing free salt there was a greater proclivity (*P* < 0.05) for professionals to include supplements in their horse(s) diet compared to non-professionals (98 versus 86%, respectively). A greater number (*P* < 0.05) of professionals fed more than two supplements compared to non-professionals (69 versus 20%, respectively). A small number of respondents (3%) fed more than 4 supplements. The majority of respondents (83%) reported to adhere to manufacturer’s guidance on feeding the various supplements; however, 12% reported to exceed the recommended levels by between 1.5 and two times the amount.

Joint supplements were the most commonly fed (22%) by all respondents, with a greater proportion (*P* < 0.05) of professionals (46%) than non-professionals (28%) including a joint supplement in their horse(s) ration. Calming supplements were fed by 13% of respondents, with more professionals than non-professionals feeding this (16% versus 11%, respectively). Digestive supplements, general vitamin and mineral supplements, and electrolyte supplements were fed the least.

The enhancement of performance (35%) and the prevention of joint disorders (34%) were the most common reasons reported by respondents for using a supplement, with a greater number of professionals (43%) feeding a supplement(s) to enhance performance compared to non-professionals (29%). A greater number (*P* < 0.05) of non-professionals (11%) also reported to provide a supplement to improve digestion compared to professionals (4%). A similar response was observed for the use of a supplement to support an underlying veterinary condition, with a greater proportion (*P* < 0.05) of non-professionals (16%) including a supplement in their horse(s) ration at times of ill health compared to professionals (8%). An overview of supplements fed by professionals and non-professionals is provided in Table 1.

**Table 1:** Overview of the percentage of non-professionals and professional respondents and their use of supplements

	Non-professionals (%)	Professionals (%)
Demographics	70^a^	30^b^
Supplement use	98^a^	86^b^
> Two supplements fed	69^a^	20^b^
Types of supplements fed:		
Salt	52^a^	25^b^
Joint supplements	46^a^	28^b^
Calming supplements	16^a^	11^a^
Performance enhancers	43^a^	29^b^
Digestive aids	11^a^	4^b^
Veterinary specific	16^a^	8^b^

Values within rows with disparate super scripts differ significantly (*P* < 0.05)

### Advice

Over 53% of respondents reported to seek advice on choosing a supplement from their feed merchant, followed by veterinary advice (46%), their trainer (34%), the internet (32%) and nutritionist (31%). A greater percentage (*P* < 0.05) of professional reported to seek advice from the internet compared to non-professionals (50 versus 26%, respectively). Books and magazines were reported as being the least common source of advice (2%) by both professionals and non-professionals. In terms of influencing factors when choosing a supplement, veterinary recommendation was given as the most influential by 90% of respondents, with no difference between professionals and non-professionals. Cost was also a major influencing factor over choice of supplement for 69% of respondents, whereas product packaging was not selected by any respondents as influencing their choice of supplement.

### Information

Over 93% of respondents reported that their understanding was that feed supplements had to meet legal standards, 72% also understood that all batches of supplement required quality testing and 92% thought that all new supplements had to be tested on horses before being launched on to the market. There were no significant differences between professionals and non-professionals in their responses to these questions. Over 80% of respondents reported that they were happy with the product information provided by supplement manufacturers; however, 64% were not satisfied with the availability of product research. The latter differed between professionals and non-professionals, with more (*P* < 0.05) professionals (70%) than non-professionals (43%) not satisfied with the amount of research conducted on products. Respondents (70%) were satisfied with the advice provided by the feed merchant selling the product; however, 82% were not satisfied with the product knowledge of the seller. A large number (89%) of respondents reported to be unsatisfied with regards to access to independent nutritional advice.

## Discussion

### Demographics

The higher number of non-professional respondents concurs with that reported in other equine-related surveys [11] as does the difference in the number of horses owned or cared for by professionals comparted to non-professionals [11].

### Supplements used

Supplement use in Ireland appears to be higher than that reported for studies conducted in the UK as a whole [6, 7] and America [1, 3, 12]. However, this current study evaluated supplement use across professional and non-professionals and all disciplines, whereas many of the previous studies have focused on a particular discipline or level of competition. The greater number of supplements fed by professionals compared to non-professionals is likely attributable to the higher demands placed on competition horses and concurs with the findings of other studies that have investigated the use of supplements in performance horses [6], where 95% of respondents reported to feed at least one supplement.

Whilst the majority of respondents reported to adhere to manufacturers’ recommended levels of feeding, over 10 % reported to exceed this with the majority of these being in the professional sector. This is contrary to National Research Council recommendations due to the risks of overdosing [13] and it would be beneficial to ascertain the rationale of owners/trainers feeding in excess of recommended levels. The most common use of joint supplements in this current study concurs with the findings of others [1], and the greater number of professionals feeding a joint supplement is in accordance with the findings of a survey of supplement use in dressage and eventing horses [6]. The higher level of use of calming supplements by professionals also concurs with Agar [6] who reported these types of supplements to be considered important for competition horses. Conversely, respondents in this current study reported electrolyte supplementation as a low priority, which is likely attributable to the high proportion of non-professionals that responded.

The reasons given for feeding supplements is in line with previous findings [1, 3, 6, 7], with the majority citing enhanced performance and prevention of joint disorders. This may be attributable to the higher number of studies conducted on joint supplementations compared to many other types of supplements, with horse owners/trainers considering joint supplements to be an important addition to their horses diet despite little evidence of their efficacy [14]. It has been proposed that owners/trainers may also be using joint supplements as a preventative measure in the development of joint problems as oppose to using them to treat a current health issue [6]. This is also an area that warrants further exploration.

Calming supplements were the second most commonly used, which again concurs with other studies [3, 6]. Behavioural issues were not specifically reported as a reason for feeding a calming supplement in the current study, although this type of supplement was cited as the second most commonly used. It is likely that this was due to the high number of non-professionals who responded to the survey as some ingredients in calming supplements are not permitted in performance horses. Other reports have highlighted behavioural issues as the most commonly reason for using a supplement [6]; however, in the same study calming supplements were not selected as most commonly used. The authors suggest that this may be attributable to a lack of availability of research on behavioural issues in horses and evidence on the efficacy of behavioural supplements [15], which may explain the findings of this current study.

### Advice and influencing factors

When looking for advice on selecting a supplement over half of respondents stated “feed merchant”, which is in contrast to previous studies who reported feed merchants to be consulted by between 10 and 15% of owners/trainers [3, 6]. However, in terms of influencing factors when choosing a supplement, veterinary recommended was given as the most influential, which concurs with other equine studies [3, 6] and humans studies where doctors/health professionals are reported to be influential in supplement choice [16]. The foregoing also fits with findings of the American Horse Publications (AHP) Equine Industry Survey 2012 whereby respondents indicated that they took advice from their veterinarian and the supplier of the supplement into account when choosing a supplement for their horse(s) [12]. Of note, was that a greater percentage of professionals used the internet for advice compared to non-professionals. The use of the internet as a source of information on equine nutrition has been reported before [3, 17], but these studies did not differentiate between sources used for supplement information versus general information on horse nutrition.

Cost significantly impacted on choice of supplement, which concurs with Agar et al. [6]; however, product packaging did not. It should not be assumed that lower cost products are more commonly selected as it has been proposed that a higher product price may influence purchase due to perceived higher quality and perceiving it as a premium product [18]. To the authors’ knowledge there have been no studies to evaluate the impact of packaging on equine supplement choice; however, studies on product packaging on consumer buying has been reported to be one of the most important factors that influences a consumer’s purchase decision [19].

### Information

A higher proportion of respondents were happy with the product information available to them in this current study compared to others [6]. Conversely, a high proportion of respondents were unhappy with the availability of product research. What was noteworthy was the high number of respondents who thought that all new supplements had to be tested on horses before being marketed as well as meeting legal standards and strict quality control. This concurs with reports on perceptions of human dietary supplements, where there appears to be significant misperceptions in consumers’ understanding of the way supplements are regulated [20]. In line with the findings of this study, the majority of consumers’ surveyed in the USA on their understanding of how human dietary supplements are regulated reported to believe that the FDA analysed all supplements before they could be sold [21]. Moreover, in excess of 40% of human physicians in the USA surveyed reported to believe that the FDA approved all dietary supplements before they can be sold to consumers [22]. The findings of this current survey of horses owners/caregivers coupled with reports on human dietary supplements suggests that there are significant misconceptions around the understanding of how supplements are tested and approved before coming to market. Moreover, there has been a lack of research on horse owners/caregivers’ beliefs on the efficacy and safety of equine supplements; however, when horse owners were asked what they believed to be the top issues facing the horse industry, they did not express concern over the safety and efficacy of equine nutritional supplements [12]. Conversely, researchers cite the lack of evidence on the safety and efficacy of equine supplements as a concern [4, 6, 7, 23].

## Conclusions

The study has highlighted the high use of supplements in the Irish equine industry, with joint supplements being the most commonly used and the reason for this being to prevent joint disorders. Feed merchants were reported as pivotal in decisions around supplement choice, but with veterinary recommendation being given as the most influential factor when decision of which supplement to buy. Of note was the misperceptions around the safety and efficacy of equine nutritional supplements, an area that warrants further exploration.
